# Reconstructing nearly isotropic microstructures to construct a one-dimensional framework causing record birefringence in thiophosphates†

**DOI:** 10.1039/d4sc03683b

**Published:** 2024-09-12

**Authors:** Lin-Tao Jiang, Xiao-Ming Jiang, Yu-Hang Fan, Bin-Wen Liu, Guo-Cong Guo

**Affiliations:** a State Key Laboratory of Structural Chemistry, Fujian Institute of Research on the Structure of Matter, Chinese Academy of Sciences Fuzhou Fujian 350002 P. R. China bwliu@fjirsm.ac.cn gcguo@fjirsm.ac.cn; b Fujian Science & Technology Innovation Laboratory for Optoelectronic Information of China Fuzhou Fujian 350002 P. R. China; c College of Chemistry, Fuzhou University Fuzhou Fujian 350116 P. R. China; d Chongqing Key Laboratory for New Chemical Materials of Shale Gas, College of Chemistry and Chemical Engineering, Yangtze Normal University Chongqing 408100 P. R. China

## Abstract

Infrared birefringent crystals that hold significant importance for optoelectronic application have been rarely reported. Traditional tetrahedral PS_4_, ethane-like P_2_S_6_, and octahedral InS_6_ units in thiophosphates typically manifest near isotropy, often resulting in extremely small birefringence. However, this study prepares α-Rb_2_InP_2_S_7_ (1), β-Rb_2_InP_2_S_7_ (2), and Cs_2_InP_2_S_7_ (3), consisting of the aforementioned microstructures, notably exhibiting the highest refractive index difference or birefringence values (0.247, 0.298, and 0.250 at 546 nm, respectively) among thiophosphates, the middle one being larger than that of commercial birefringent materials. This unusual increase in birefringence can be primarily attributed to two key factors: (1) simultaneous stretching and compressing of the P–S and In–S covalent bond interactions, generating high polarizability anisotropy of InS_6_, PS_4_, and P_2_S_6_ polyhedral units; (2) the additional incorporation of alkali metals that further reduces the dimensionality of the crystal structure, creating one-dimensional [InP_2_S_7_]^2−^ structures with increasing polarizability anisotropy. This study presents an alternative approach to enhance birefringent materials by reconstructing covalent bond interactions and specific spatial arrangements.

## Abstract 2

Novel birefringent materials, α-Rb_2_InP_2_S_7_, β-Rb_2_InP_2_S_7_, and Cs_2_InP_2_S_7_ were reconstructed from the nearly isotropic basic building units and displayed the largest birefringence among all known thiophosphates.

## Graphical abstract

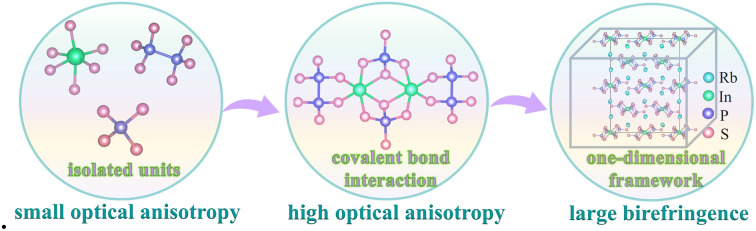

## Introduction

Birefringence, which refers to the difference in refractive indices for different polarizations of light in anisotropic crystals, finds widespread applications in optics, such as light polarization, optical modulation, and nonlinear optical materials.^1–19^ To fulfill the technical demands of miniaturized designs, materials must exhibit substantial birefringence. In recent decades, several materials with remarkable birefringence have been discovered and commercially developed, including α-BaB_2_O_4_ (0.122 at 532 nm),^20^ LiNbO_3_ (0.074 at 1300 nm),^21^ YVO_4_ (0.204 at 1064 nm),^22^ and TiO_2_ (0.256 at 1530 nm).^23^ These oxide crystals are restricted to the ultraviolet, visible or near-infrared (IR) wavelength ranges due to their IR cut-off region there, thereby, for the IR region, limiting progress in the development of non-oxide crystals, such as phosphides, sulfides, and halides. For example, Niu studied the quasi-one-dimensional (1D) crystal BaTiS_3_, where the perovskite octahedral [TiS_6_] units are arranged in parallel chains, exhibiting a significantly large birefringence coefficient of 0.76.^2^ Moreover, due to their sensitivity to air or challenges in crystal growth, viable bulk crystals suitable for device applications in the IR region are very limited.

Thiophosphates, as a subclass of chalcogenides, have garnered significant attention due to their diverse structures.^24,25^ However, their development as birefringent materials has been hindered by the limited optical anisotropy associated with the basic units [PQ_4_]^3−^ (Q = S, Se, Te) and ethane-like [P_2_Q_6_]^4−^. To address this challenge, the incorporation of structurally favorable polarization anisotropy units has proven beneficial. Traditionally, increased birefringence has been achieved by various additional building blocks, including those with Jahn–Teller effects and stereochemically active lone pair electrons in metal-centered polyhedral, π-conjugated planar triangular units, and mixed-anion units. This approach has been successfully applied to several thiophosphates. For instance, TlBiP_2_S_6_ and KSbP_2_S_6_, which feature stereochemically active lone pair [BiS_3_] or [SbS_3_] trigonal pyramidal units, exhibit enhanced birefringence (calculated: 0.155 at 2090 nm and 0.162 at 2090 nm, respectively).^26,27^ Additionally, the introduction of a mixed anion [ZnS_2_I_2_] structural unit has led to a chalcohalide, Cs_4_Zn_5_P_6_S_18_I_2_, displaying significant birefringence (experimental: 0.128 at 546 nm).^23^ However, to the best of our knowledge, no thiophosphates with high experimental birefringence have been achieved beyond the range of the polarization anisotropy structural units mentioned above.

**Fig. 1:**
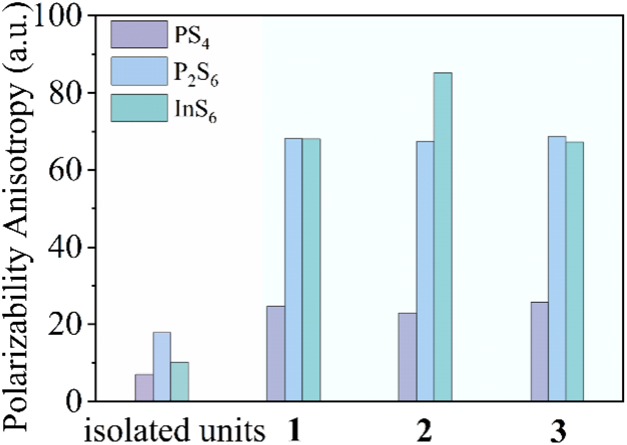
The calculated static polarizability anisotropy of structural units (isolated tetrahedral PS_4_ in Li_3_PS_4_,^30^ ethane-like P_2_S_6_ in Li_4_P_2_S_6_,^31^ octahedral InS_6_ in Y_3_InS_6_,^32^ and compounds 1–3).

Group IIIA elements are widely utilized in compound synthesis and have unique properties due to their diverse coordination environments, such as typical tetrahedral or octahedral configurations.^28,29^ When incorporating octahedral InQ_6_ units into a thiophosphate, as depicted in Fig. 1, they often result in near polarizability isotropy, which can reduce birefringence. Yet, to analyze a special type of chemical bonding, it is observed that if a chemical P–Q bond can form an additional linkage with the InQ_6_ bond, with varying degrees of stretching or compressing, it subsequently affects the P–Q bonds of adjacent chemical bonds. This interaction leads to the formation of highly polarized octahedral InQ_6_, tetrahedral PQ_4_, or ethane-like P_2_Q_6_ units. Meanwhile, the introduction of electropositive alkali metal atoms can induce a dimensional reduction in the crystal structure, thereby lowering its dimensionality, which typically leads to larger birefringence.^2,33,34^ Therefore, such changes in bond distances, angles, and dimensions can significantly enhance the polarizability anisotropy of micro- and macro-structures, offering an alternative approach to effectively manipulate birefringence.

Guided by the aforementioned ideas, the element In is incorporated into thiophosphate, resulting in the construction of 1D structures separated by alkali metal cations Rb^+^ and Cs^+^. These compounds, namely α-Rb_2_InP_2_S_7_ (1), β-Rb_2_InP_2_S_7_ (2), and Cs_2_InP_2_S_7_ (3), display the largest reported refractive index difference or birefringence (0.247, 0.298, and 0.251 at 546 nm, respectively) among thiophosphates. This significant refractive index difference or birefringence is primarily attributed to the coupling of distorted InS_6_, P_2_S_6_, and PS_4_ polyhedrons with anisotropic polarizability through stretching and compressing covalent P–S and In–S bonds to build the 1D structures, enhancing the anisotropic polarizability and finally increasing the birefringence. Moreover, the large birefringence plays a crucial role in the phase-matching ability of compound 2, regarded as a second-order nonlinear optical material.

## Results and discussion

Compounds 1–3 were synthesized using a high-temperature solid-phase reaction-assisted flux method, as described in the ESI.† The semiquantitative elemental compositions of crystals 1–3 were confirmed through energy-dispersive X-ray spectroscopy (EDS) analysis, which closely matched the chemical formulas determined by single-crystal X-ray diffraction (XRD; Fig. S1†). It is important to observe that, while the simulated XRD patterns for compounds 1 and 2 bear a striking resemblance, subtle shifts in their three prominent peaks are discernible, effectively differentiating the two, as shown in Fig. S2.† So, the purity analysis conducted *via* powder XRD confirmed that samples 1–3 were composed of pure phases. The IR spectra showed that compounds 1–3 exhibited transmission ranges from 2.5 to 14.3 μm, indicating coverage of the two key atmospheric windows (3–5 μm and 8–14 μm; Fig. S3a†). The optical absorption spectra revealed optical band gaps of 3.23, 3.19, and 3.24 eV for compounds 1–3, respectively (Fig. S3b†). The electronic band structures, as displayed in Fig. S4a–c,† indicated direct band gaps of 2.03, 2.01, and 2.02 eV for compounds 1–3, respectively. Additionally, the partial density of states (Fig. S4d–f†) analysis indicated that the minimum conductive bands were primarily determined by In-5s, P-3s/3p, and S-3p states, while the maximum valence bands were mainly composed of S-3p states. Therefore, their band gap transitions are principally determined by the contribution of anionic groups. These wide band gaps effectively prevent thermal effects caused by two-photon absorption under 1064 nm laser irradiation, one of the main reasons for the higher laser-induced damage threshold.

**Fig. 2:**
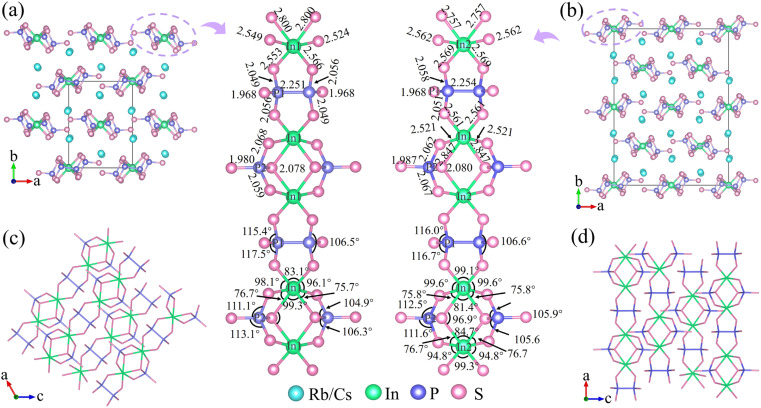
The crystal structures of compounds 1 (a) and 2 (b) are each viewed along the *c* axis, and the In and P elements are marked. 1D [InP_2_S_7_]^2−^ anionic chains are perpendicular to the *b* axis for 1 (c) and 2 (d).

Single-crystal XRD analyses revealed that compounds 1–3, although having the same stoichiometry, crystallize in different space groups: monoclinic *P*2_1_/*n*, orthorhombic *Fdd*2, and monoclinic P2_1_/*n*, respectively (Tables S2 and S3†). Thermal analysis confirmed that phases 1–3 do not undergo phase transitions with temperature changes (Fig. S5†). The crystal structures of 1 and 2 are illustrated in Fig. 2, and all phases exhibit 1D [InP_2_S_7_]^2−^ anionic chains extending along different directions, with alkali metal cations embedded to stabilize the frameworks and maintain charge balances. The 1D [InP_2_S_7_]^2−^ chains are constructed of ethane-like [P_2_S_6_]^4−^, tetrahedral [PS_4_]^3−^, and octahedral [InS_6_]^9−^ units. All In atoms are coordinated by six S atoms to form octahedral [InS_6_]^9−^ building blocks, which are connected through edge-sharing S atoms to two isolated [PS_4_]^3−^ units, yielding the [InP_2_S_12_]^11−^ units. Each [InP_2_S_12_]^11−^ unit further connects with others through ethane-like [P_2_S_6_]^4−^ units to construct the [InP_2_S_7_]^2−^ chains. The spatial arrangements of the anionic frameworks differ due to the distinct coordination environments of the cations. Thus, compounds 1 and 3 possess 2 screw axes and n glide planes, while compound 2 has a 2-fold axis, resulting in structural polymorphism among the crystals.

As illustrated in Fig. 2, in these structures, two octahedral [InS_6_]^9−^ units are adjacent with two shared S atoms, producing the [In_2_S_10_] dimer. The tetrahedral [PS_4_]^3−^ units connect to the [InS_6_]^9−^ dimer through two terminal and one bridging [In_2_S_10_] dimer, resulting in elongated In–S bonds (2.800 and 2.847 Å for 1 and 2, respectively) and large bond angle changes (105.6–112.5°). The ethane-like [P_2_S_6_]^4−^ units are attached to the InS_6_ unit *via* two terminal protruding vertices, resulting in short bonds (2.561 and 2.569 Å) and small bond angle changes (75.7° and 75.8°). Compared to regular octahedral InS_6_ units with equal bond lengths (∼2.5 Å) and angles (90°), these distorted octahedral InS_6_ units exhibit significant distortions, with bond distances ranging from 2.521 to 2.847 Å and S–In–S bond angles ranging from 75.8° to 112.5° (Tables S4 and S5†). Similarly, the two tetrahedral PS_4_ units with bond distances of 1.987–2.080 Å and bond angles of 75.8–112.5°, as well as the two ethane-like P_2_S_6_ units with a bond distance of 2.254 Å and bond angles of 106.6–116.7°, also undergo severe distortions. Therefore, the covalent bonding between the P–S (or In–S) bonds exerts different directional tensions on the In–S (or P–S) bonds, leading to the distortion of octahedral InS_6_, tetrahedral PS_4_, and ethane-like P_2_S_6_ units. Additionally, the degree of distortion in the tetrahedral PS_4_, ethane-like P_2_S_6_, and octahedral InS_6_ units of compounds 1–3 was determined by Lalik's distortion index (Δ*H*) and Brown's distortion index (Δ*R*).^35–37^ As detailed in Table S1,† the values of Δ*H* and Δ*R* for the InS_6_ octahedra are notably higher compared to those for the PS_4_ and P_2_S_6_ units. By comparing the polarizability anisotropy of the isolated and covalently bonded InS_6_, P_2_S_6_, and PS_4_ units (10.1, 17.8, 10.1 for isolated units; 67.3, 68.7, 25.8 for 1; 85.2, 67.4, 22.9 for 2; and 68.1, 68.1, 24.6 for 3; respectively (Fig. 1)), it can be observed that the structures exhibit significant polarizability anisotropy after covalent bonding. Furthermore, the S atoms form 8 to 10 coordination bonds with alkali atoms, resulting in bond lengths ranging from 3.349 to 3.965 Å (Fig. S6†). As expected, the introduction of cations leads to the formation of 1D structures in compounds 1–3. This occurrence would improve the optical anisotropy of charge distribution along parallel and perpendicular directions, which typically display higher birefringence.

**Fig. 3:**
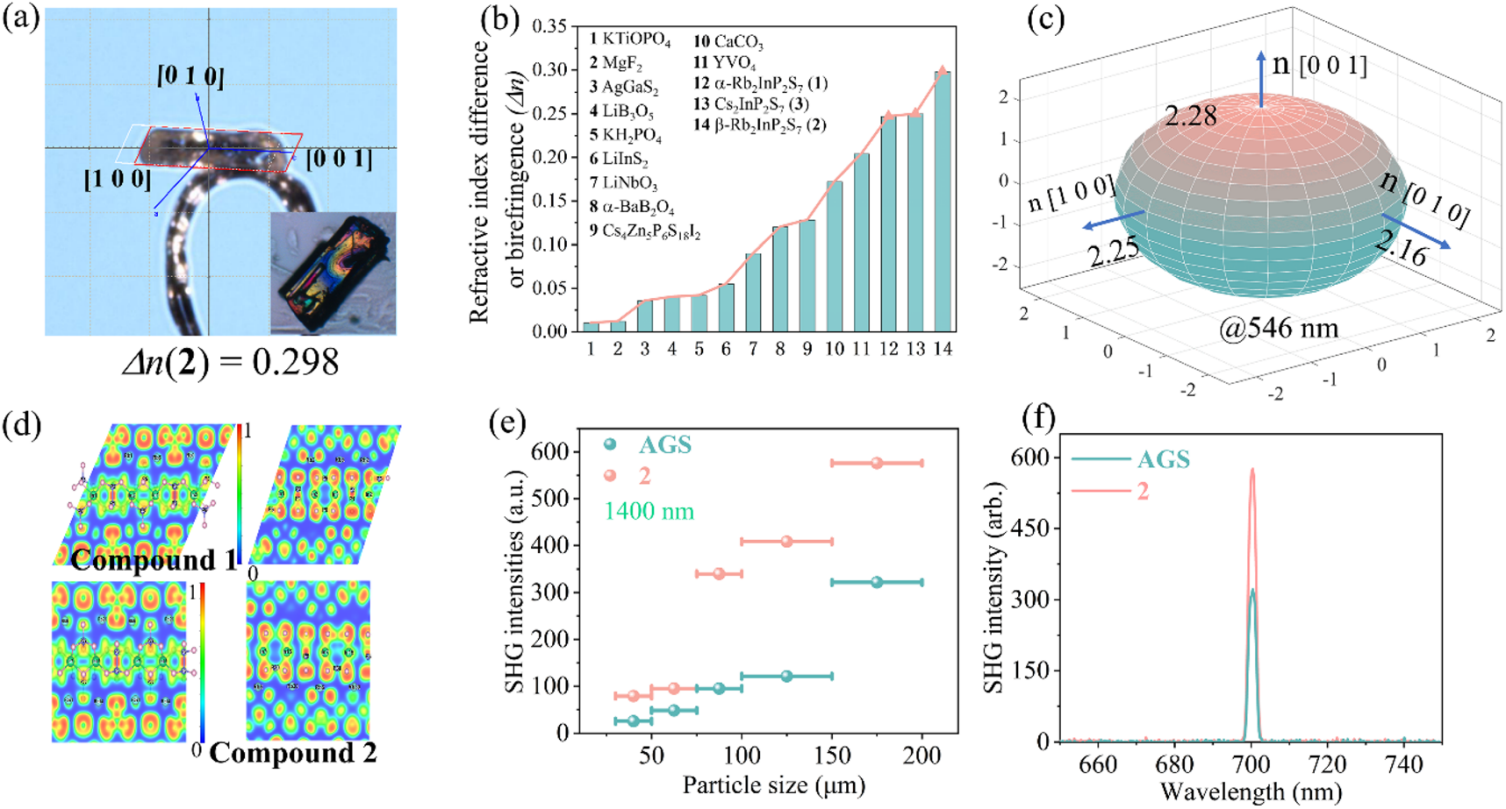
(a) The crystal orientation of 2 is determined by single-crystal XRD. (b) Comparison of experimental refractive index difference or birefringence of 1–3 and commercial birefringent materials. (c) Theoretically calculated refractive indices of compound 2. (d) The 2D slice of electron localization function for crystals 1 and 2. The phase-matchable behavior (e) and relative SHG intensities (f) of compound 2 and AgGaS_2_ at 1400 nm.

To determine the birefringence of these crystals, single-crystal XRD was performed to determine the crystal orientation. As shown in Fig. 3a and S7,† the crystal planes indexed for crystals 1–3 were (0 0 1), (0 1 0), and (0 0 1), respectively. Then, colorless crystals of compounds 1–3 were observed under a Berek compensator with 546 nm incident polarized light. The refractive index difference or birefringence was measured using a polarizing microscope, which yielded optical path difference (*R*) and crystal thickness (*T*) values of 13.6, 11.9, and 12.5 μm, and 55, 40, and 50 μm, respectively. The experimentally determined refractive index difference or birefringence values were calculated using the formula *R* = |*n*_e_ – *n*_o_| × *T*.^35^ The refractive index difference or birefringence values were found to be 0.247, 0.298, and 0.250 (at 546 nm), respectively, which are the largest among the reported thiophosphates. Thus, due to the monoclinic crystal class of compounds 1 and 3, the experimental values obtained from the crystal plane indices (0 0 1) accurately reflect the refractive index difference. In the case of compound 2, which is orthorhombic, these indices truly represent birefringence. Crystals 1–3 displayed significantly higher refractive index difference or birefringence compared to commercial oxide birefringent materials (Fig. 3b). The calculated birefringence values for compounds 1 and 3 are revealed in Fig. S7c and d,† with the order of the three refractive indices being *n*_*y*_ > *n*_*z*_ > *n*_*x*_, and the calculated birefringence values are 0.11 and 0.10 (at 546 nm), respectively. For phase 2, as depicted in Fig. 3c, there are three unequal principal refractive indices, with *n*_*z*_ > *n*_*y*_ > *n*_*x*_ along the principal optical axis. Therefore, birefringence can be defined as *n*_*z*_ − *n*_*x*_, and the calculated birefringence value is 0.13 (at 546 nm). Additionally, electron localization function (ELF) maps of compounds 1 and 2 were calculated by projecting onto crystal planes parallel to the [InP_2_S_7_]^2−^ chains (Fig. 3e).

The electron distribution, which is more uniformly distributed along the linear molecules, increases the anisotropy of the charge distribution in the directions parallel and perpendicular to the molecule direction. Consequently, it is visually apparent that there are substantial variations in electron density between the vertical and horizontal crystal chains, resulting in strong optical polarizability anisotropy.

Furthermore, compound 2 possesses a noncentrosymmetric structure, making it suitable for nonlinear optical-active materials. Generally, an optical anisotropic crystal can utilize its birefringence to counteract the impact of dispersion, thus realizing phase-matching capability. Fig. 3e and f and S8† demonstrated that the doubling intensity of phase 2 was directly proportional to the crystal size within a broadband laser wavelength range of 1064–1600 nm, indicating phase-matching behavior. The experimental results revealed that under laser wavelengths of 1064, 1200, 1300, 1400, 1500, and 1600 nm, the second-harmonic generation (SHG) intensities of compound 2 were 6.4, 2.7, 1.9, 1.7, 0.4, and 0.3 times that of benchmark AgGaS_2_, respectively. These values were comparable to the SHG responses observed in AgHg_3_PS_6_ (0.5 × AgGaS_2_),^36^ LiZnPS_4_ (0.8 × AgGaS_2_),^37^ and [K_3_Br][Ga_3_PS_8_] (1.2 × AgGaS_2_).^38^ The significant birefringence exhibited by compound 2 is a crucial factor in achieving phase-matching behavior, thereby facilitating its application as an excellent nonlinear optical material.

## Conclusions

In summary, although thiophosphates constructed by the normal octahedral InQ_6_ unit typically exhibit low polarizability anisotropy and very small birefringence, this study successfully synthesized three thiophosphates, 1–3, using a solid-state reaction method and the experimental refractive index difference or birefringence values for compounds 1–3 are equal to 0.247, 0.298, and 0.250 (at 546 nm), respectively, the middle one is the highest birefringence among all known thiophosphates. The rational reason is that the formation of one-dimensional [InP_2_S_7_]^2−^ chains through enhanced interactions between the covalent In–S and P–S bonds results in strong polarizability anisotropy. Furthermore, compound 2 demonstrates remarkable phase-matching capabilities in terms of SHG intensity, indicating its potential applications as an outstanding nonlinear optical material. This study highlights the effectiveness of achieving birefringent crystals through the interaction of microstructural units with specific spatial arrangements.

## Experimental

All reagents, barium metal (99.9%, Aladdin), indium powder (99.9%, Aladdin), red phosphorus powder (99.9%, Aladdin), sulfur powder (99.9%, Sinopharm), ACl (A = Rb and Cs) powder (99.9%, Aladdin), were stored and handled within an argon-filled glovebox. For synthesis of 1: barium (0.37 mmol), indium (0.37 mmol), phosphorus (0.75 mmol), sulfur (2.59 mmol), and RbCl (0.45 mmol); for 2: barium (0.37 mmol), indium (0.37 mmol), phosphorus powder (0.75 mmol), sulfur (2.59 mmol), and RbCl (1.24 mmol); for 3: barium (0.37 mmol), indium (0.37 mmol), phosphorus (0.75 mmol), sulfur (2.59 mmol), and CsCl powder (0.94 mmol); all raw materials were accurately weighed and mixed. The resulting mixtures were loaded into quartz tubes, which were then sealed under a vacuum of approximately 10^−3^ torr using a hydrogen-oxygen flame. The quartz tubes were placed in a computer-controlled muffle furnace. For 1, the reaction was heated to 973 K over 24 hours, and maintained at that temperature for 96 hours before being gradually cooled to 573 K over 96 hours. For 2, the reactions were heated to 1023 K over 24 hours, and kept at this temperature for 96 hours before being gradually cooled to 573 K over 96 hours. Compounds 1 and 2 are isomers and altering the molar ratios of metal salts, such as RbCl, along with the temperature conditions, can result in two distinct phases. For 3, the mixture was heated to 1023 K in 24 hours, held at that temperature for 96 hours, and gradually cooled to 573 K over 96 hours. Finally, the furnace was allowed to naturally cool to room temperature by switching off the power. The elemental compositions of crystals 1–3 were confirmed using EDS combined with a Hitachi S-3500 scanning electron microscope. High-quality crystals of 1–3 were carefully selected for single-crystal XRD measurements using a Rigaku Pilatus CCD diffractometer with graphite-monochromatic Mo-Kα radiation (*λ* = 0.71073 Å) at 293 K.^39^ The intensity data were collected using a ω-scan technique and subsequently processed using CrysAlisPro software.^40^ The crystal structures were solved using direct methods and refined using full-matrix least-squares methods on *F*^2^, incorporating anisotropic thermal parameters for all atoms with the Shelxtl package.^41^ Additional symmetry of the final structures was examined using the Addsym/Platon program,^42^ which confirmed the absence of any higher symmetry elements. The powder XRD patterns of carefully selected crystalline samples of 1–3 were obtained using a Rigaku Mini Flex 600 X-ray diffractometer equipped with a diffracted monochromator set for Cu-Kα radiation (*λ* = 1.54057 Å) at 30 kV and 40 mA. The thermal properties of phases 1–3 were investigated using a differential scanning calorimeter (DSC) with a TGA/DSC Mettler Toledo thermal analyzer. Polycrystalline samples weighing 10.0 mg for compounds 1–3 were enclosed in sealed silica tubes under vacuum conditions. Two heating and cooling cycles were performed, each reaching a maximum temperature of 800 °C at a rate of 10 °C min^−1^. The DSC curves revealed distinct endothermic peaks at temperatures of 590.1, 589.6, and 596.5 °C, and exothermic peaks were observed at temperatures of 456.1, 467.4, and 464.3 °C for phases 1 and 3, respectively. The residues obtained before and after the DSC measurements were found to be inconsistent, suggesting that all phases do not undergo congruent melting processes. The optical diffuse reflectance of phases 1–3 was measured using a PerkinElmer Lambda 900 UV-vis spectrophotometer at room temperature. The measurement wavelength ranged from 200 to 2500 nm, with KBr serving as a 100% reflectance standard. The absorption spectra were obtained from the reflection spectra using the Kubelka–Munk formula: *α*/*S* = (1 *R*)^2^/2*R*, where *α* represents the absorption coefficient, *S* is the scattering coefficient, and *R* is the reflectance.^43^ Additionally, IR spectra of powdered samples of 1–3 were obtained using a Nicolet Magana 750 FT-IR spectrophotometer. The birefringence of crystals 1–3 was characterized by using a polarizing microscope (Nikon Eclipse LV100N POL) equipped with a Berek compensator.^44^ Transparent strip-like crystals 1–3 were chosen for measurements to improve the accuracy of the refractive index difference or birefringence under the wavelength of 546 nm of the light source. Powder SHG response of 2 was investigated using a modified Kurtz and Perry method using an Andor DU420A-BR-DD CCD camera,^45^ with laser radiation ranging from 1064 to 1600 nm, at room temperature. To perform phase-matching measurements, compound 2 and AgGaS_2_ samples were divided into different particle sizes, specifically within the ranges of 30–50, 50–75, 75–100, 100–150, and 150–200 μm. The measurements were conducted using lasers operating at wavelengths of 1064, 1200, 1300, 1400, 1500, and 1600 nm. Moreover, the static polarizability anisotropy of structural units in PS_4_, P_2_S_6_, and InS_6_ polyhedra was examined using the Gaussian 09 package with the PBE1PBE functional method and def2TZVP basis set.^46^ Further, the electron localization function diagrams of 1 and 2 in the *ac* plane can be visualized using VESTA software.^47^ The electronic band structures of 1–3 were calculated using the ABINIT software package, which is based on density functional theory (DFT).^48,49^ The exchange-correlation function was chosen as the generalized gradient approximation, and projector-augmented plane-wave pseudopotentials were employed with the following valence configurations: Rb-4s^2^4p^6^5s^1^, Cs-5s^2^5p^6^6s^1^, In-5s^2^5p^1^4d^10^, P-3s^2^3p^3^, and S-3s^2^3p^4^. The plane-wave energy cutoff value was set to 18 hartree. For the Brillouin zone numerical integration, a 3 × 3 × 3 Monkhorst–Pack *κ*-point grid was used for 1 and 3, while a 3 × 3 × 2 grid was used for 2. The birefringence of compounds 1–3 was calculated by Δ*n* = max |*n*_*i*_ − *n*_*j*_| *i*, *j* = 1, 2, 3; *i* ≠ *j*. The refractive index (*n*) was obtained using the following formula:^50^
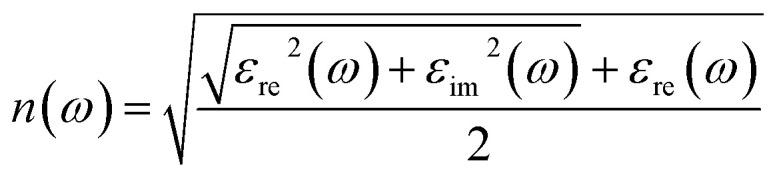


## Author contributions

L.-T. Jiang and B.-W. Liu were responsible for the conception and design of the experiments. L.-T. Jiang drafted the initial manuscript. X.-M. Jiang and Yu-Hang Fan conducted the theoretical calculations. B.-W. Liu and G.-C. Guo contributed to revising the manuscript. All authors collaborated on the preparation of the final manuscript.

## Conflicts of interest

The authors declare no competing financial interest.

## Supplementary Material

SC-015-D4SC03683B-s001

SC-015-D4SC03683B-s002

## Data Availability

All of the related experimental and computational data are provided in the ESI.†
