# Supplementary material for: Reconstructing nearly isotropic microstructures to construct a one-dimensional framework causing record birefringence in thiophosphates
Source: Chem Sci. 2024 Sep 12;15(41):17114–9. doi: 10.1039/d4sc03683b (PMC11440415; doi:10.1039/d4sc03683b)
Supplement: SC-015-D4SC03683B-s001 [file SC-015-D4SC03683B-s001.pdf]

Supplementary Information (SI) for Chemical Science.
This journal is © The Royal Society of Chemistry 2024

Supporting Information

Reconstructing Nearly Isotropic Microstructures to Construct One-dimensional Framework Causing the Record Birefringence in Thiophosphates

Lin-Tao Jiang,[a,c] Xiao-Ming Jiang,[a,b] Yu-Hang Fan,[d] Bin-Wen Liu,[a,b,] * and Guo-Cong Guo[a,b,] *

[a] *State Key Laboratory of Structural Chemistry, Fujian Institute of Research on the Structure of Matter, Chinese Academy of Sciences, Fuzhou, Fujian 350002, P. R. China*

[b] *Fujian Science & Technology Innovation Laboratory for Optoelectronic Information of China, Fuzhou, Fujian 350108, P. R. China*

[c] *College of Chemistry, Fuzhou University, Fuzhou, Fujian 350116, P. R. China*

[d] *Chongqing Key Laboratory for New Chemical Materials of Shale Gas, College of Chemistry and Chemical Engineering, Yangtze Normal University, Chongqing 408100, P. R. China*

**Corresponding Authors: bwliu@fjirsm.ac.cn; gcguo@fjirsm.ac.cn*

Contents

1. Tables

Table S1. Crystallographic data and structure refinement results of **1**-**3**.

Table S2. The calculated Lalik's distortion index (ΔH) and Brown's distortion index (ΔR) of asymmetric tetrahedral and octahedral units in **1**-**3**.

Table S3. Fractional atomic coordinates, equivalent isotropic displacement parameters, formal oxidation states and bond valence sum of all atoms in **1**-**3**.

Table S4. Selected bond lengths of **1**-**3**.

Table S5. Selected bond angles of **1**-**3**.

2. Figures

Fig. S1 EDS spectra of compounds **1**-**3**.

Fig. S2 Powder XRD patterns of **1**-**3**.

Fig. S3 IR spectra and Band gaps of **1**-**3**.

Fig. S4 Electronic band structures and the partial DOS of **1**-**3**.

Fig. S5 DSC cycle of **1**-**3**.

Fig. S6 Coordination environment diagram of Rb/Cs in **1**-**3**.

Fig. S7 The crystals orientation of **1** (a) and **3** (b) are determined by single-crystal XRD. Theoretically calculated refractive indices and birefringence of **1** (c) and **3** (d).

Fig. S8 (a-e) Phase-matchable behaviors of compound **2** and $AgGaS_2$. (b) Broadband SHG intensities of **2** and $AgGaS_2$.

1 Tables

Table S1. Crystallographic data and structure refinement results of **1**-**3**.

Empirical formula	α-$Rb_2InP_2S_7$ (**1**)	β-$Rb_2InP_2S_7$ (**2**)	$Cs_2InP_2S_7$ (**3**)
*F*w	572.12	572.12	667.00
Temperature (K)		298	
Space group	$P2_1/n$	*Fdd*2	$P2_1/n$
a (Å)	10.7564(5)	18.6758(5)	10.9132(5)
b (Å)	12.6856(3)	25.4262(6)	13.1589(4)
c (Å)	10.7941(5)	10.7974(2)	10.9640(5)
α (°)	90	90	90
β (°)	119.599(6)	90	119.511(6)
γ (°)	90	90	90
Volume ($Å^3$)	1280.7(1)	5127.2(2)	1370.2(1)
Z	4	16	4
ρ_{calc} (g/cm^3)	2.967	2.965	3.233
μ (mm^{-1})	10.738	10.727	8.213
F(000)	1060.0	4240.0	1204.0
Data/restraints/parameters	2874/0/109	2211/1/111	2322/0/109
Flack parameter	/	0.0604(5)	/
GOF on F^2	0.999	1.001	1.001
$R_1{}^a$ ($I > 2\sigma(I)$)	0.0399	0.0152	0.0356
$wR_2{}^b$ ($I > 2\sigma(I)$)	0.0969	0.0384	0.0955
$R_1{}^a$ (all data)	0.0485	0.0159	0.0385
$wR_2{}^b$ (all data)	0.1005	0.0386	0.0972
$\Delta\rho_{max}/\Delta\rho_{min}$ (e $Å^{-3}$)	2.00/-1.10	0.86/-0.35	2.74/-0.92

$^aR = \Sigma||F_o|-|F_c||/\Sigma|F_o|$, $^bwR = (\Sigma(w(F_o{}^2-F_c{}^2)^2)/\Sigma(w(F_o{}^2)^2))^{1/2}$.

Table S2. The calculated Lalik's distortion index (ΔH) and Brown's distortion index (ΔR) of asymmetric tetrahedral and octahedral units in **1**-**3**.

Compounds	units	ΔH	ΔR
α-$Rb_2InP_2S_7$ (**1**)	PS_4	0.008424	0.002090
	P_2S_6	0.017454	0.004384
	InS_6	0.062340	0.017724
β-$Rb_2InP_2S_7$ (**2**)	PS_4	0.007501	0.001865
	P_2S_6	0.017434	0.002190
	In(1)S_6	0.087474	0.025573
	In(2)S_6	0.037280	0.010311
$Cs_2InP_2S_7$ (**3**)	PS_4	0.006826	0.001697
	P_2S_6	0.012488	0.006250
	InS_6	0.083017	0.024103

Table S3. Fractional atomic coordinates (×10^4), equivalent isotropic displacement parameters (Å^2×10^3), formal oxidation states (FOS) and bond valence sum (BVS) of all atoms in **1**–**3**.

α-$Rb_2InP_2S_7$ **(1)**						
Atom	*x*	*y*	*z*	*U*(eq)	FOS	BVS
Rb1	5065.9(7)	1681.7(4)	4622.4(7)	34.6 (1)	1	0.860
Rb2	5184.2(7)	1546.9(5)	456.6(7)	58.8(1)	1	0.856
In1	9997.2(4)	33.1(2)	3056.4(4)	19.7(1)	3	3.099
P1	1188.3	9859.5(9)	577.3(1)	15.7(1)	4	4.184
P2	7390.9(1)	9321.1(9)	3724.5(1)	18.2(3)	5	5.253
S1	8340.4 (3)	801.8(9)	4203.5(1)	22.3(3)	-2	-1.824

S2	1786.7 (3)	1476.9(9)	4369.9(1)	22.0(3)	-2	-2.165
S3	1514.5 (2)	8948.2(9)	2287.0(1)	21.6(3)	-2	-2.073
S4	8555.1(1)	1115.6(9)	793.1(1)	22.2(3)	-2	-2.021
S5	8103.8(1)	8616.8(9)	2462.0(1)	22.3(3)	-2	-2.175
S6	2207.0(1)	1216.8(1)	1057.4(1)	25.8(3)	-2	-1.961
S7	4722.0 (8)	580.2(1)	7287.8(1)	30.9(3)	-2	-2.033

β-$Rb_2InP_2S_7$ **(2)**

Atom	*x*	*y*	*z*	*U*(eq)	FOS	BVS
Rb1	2466.7(3)	9148.7(2)	6781.9(5)	38.2(1)	1	0.776
Rb2	2587.9(3)	756.8(2)	7537.2(5)	38.6(1)	1	0.883
In1	0	0	2710.5(4)	21.8(1)	3	3.073
In2	0	0	6589.9(4)	22.6(1)	3	3.062
P1	4404.9(5)	69.6(4)	4662.7(1)	18.3(1)	4	4.157
P2	1310.2(5)	344.0(4)	4682.4(1)	20.8(1)	5	5.211
S1	832.7(5)	9603.0(4)	4705.9(3)	25.0(1)	-2	-1.952
S2	886.9(6)	721.4(5)	3155.2(1)	26.0(2)	-2	-2.070
S3	731.5(6)	9454.7 (5)	1174.5(1)	24.9(2)	-2	-2.169
S4	2639.0(5)	9703.2(5)	9635.7(1)	33.8(3)	-2	-1.952
S5	946.7(6)	714.2 (5)	6269.1(9)	24.4(2)	-2	-1.819
S6	753.8(6)	9466.5(5)	8128.2(1)	23.9(2)	-2	-2.135
S7	3894.6(5)	9392.5(4)	4678.6(1)	27.5(2)	-2	-1.998

$Cs_2InP_2S_7$ (**3**)

Atom	*x*	*y*	*z*	*U*(eq)	FOS	BVS
Cs1	4647.2(6)	1695.7(4)	137.6(5)	35.43(1)	1	1.098
Cs2	517.4(5)	1573.1(4)	254.5(6)	39.81(1)	1	1.047
In1	3040.9(5)	53.2(4)	4991.1(5)	25.66(1)	3	3.032
P1	6278.1(1)	614.6(1)	7577.7(1)	22.1(1)	4	4.146
P2	9430.0(1)	154.9(1)	3847(2)	24.9(4)	5	5.232

S1	7271.5(1)	536.2(1)	9657.6(1)	32.5(5)	-2	-2.139
S2	4408.6(1)	1401.8(1)	6784.8(1)	25.8(4)	-2	-2.206
S3	7559.4(1)	1269.6(1)	6899.7(1)	26.7(4)	-2	-2.244
S4	4221(2)	803.1(1)	3372(2)	28.2(4)	-2	-1.767
S5	2724.9(1)	3985.1(1)	8546(2)	30.0(4)	-2	-2.111
S6	1035(2)	1118.5(1)	7219(2)	34.6(5)	-2	-2.044
S7	796(2)	1132.7(1)	3643(2)	32.7(5)	-2	-2.044

Table S4. Selected bond lengths (Å) of **1**-**3**.

α-$Rb_2InP_2S_7$ (**1**)					
bond	length/Å	bond	length/Å	bond	length/Å
Rb1–S1	3.965(1)	Rb2–S2	3.625(1)	In2–S5	2.562(1)
Rb1–S1	3.928(1)	Rb2–S1	3.798(1)	In1–S4	2.553(1)
Rb1–S2	3.411(1)	Rb2–S2	3.554(1)	In1–S5	2.550(1)
Rb1–S3	3.456(1)	Rb2–S4	3.504(1)	P1–P1	2.251(1)
Rb1–S3	3.630(1)	Rb2–S5	3.387(1)	P1–S3	2.056(1)
Rb1–S4	3.754(1)	Rb2–S5	3.354(1)	P1–S4	2.049(1)
Rb1–S6	3.595(1)	Rb2–S6	3.592(1)	P1–S6	1.968(1)
Rb1–S6	3.360(1)	Rb2–S7	3.602(1)	P2–S1	2.078(1)
Rb1–S7	3.606(1)	In1–S1	2.800(1)	P2–S2	2.059(1)
Rb1–S7	3.379(1)	In1–S2	2.524(1)	P2–S5	2.068(1)
Rb2–S1	3.900(1)	In1–S3	2.566(1)	P2–S7	1.980(2)
β-$Rb_2InP_2S_7$ (**2**)					
Bond	Length/Å	Bond	Length/Å	Bond	Length/Å
Rb1–S1	3.965(1)	Rb2–S2	3.625(1)	In2–S5	2.562(1)
Rb1–S2	3.435(1)	Rb2–S3	3.514(1)	In2–S6	2.570(1)
Rb1–S2	3.759(1)	Rb2–S4	3.376(1)	P1–P1	2.251(1)
Rb1–S4	3.409(1)	Rb2–S4	3.515(1)	P1–S3	2.051(1)

Rb1–S4	3.738(1)	Rb2–S5	3.364(1)	P1–S6	2.059(1)
Rb1–S6	3.611(1)	Rb2–S5	3.349(1)	P1–S7	1.971(1)
Rb1–S6	3.433(3)	Rb2–S7	3.633(1)	P2–S1	2.080(1)
Rb1–S7	3.351(1)	In1–S1	2.847(1)	P2–S2	2.062(1)
Rb1–S7	3.563(1)	In1–S2	2.521(1)	P2–S4	1.987(1)
Rb2–S1	3.852(1)	In1–S3	2.561(1)	P2–S5	2.067(1)
Rb2–S1	3.882(1)	In2–S1	2.757(1)		

$Cs_2InP_2S_7$ (**3**)					
Bond	Length/Å	Bond	Length/Å	Bond	Length/Å
Cs1–S1	3.506(2)	Cs2–S2	3.658(2)	In1–S5	2.562(2)
Cs1–S1	3.677(2)	Cs2–S3	3.523(1)	In1–S7	2.576(2)
Cs1–S2	3.576(1)	Cs2–S3	3.510(2)	P1–P1	2.237(4)
Cs1–S4	3.957(2)	Cs2–S4	3.912(2)	P1–S6	1.962(3)
Cs1–S5	3.594(2)	Cs2–S4	3.929(2)	P1–S5	2.061(3)
Cs1–S5	3.699(1)	Cs2–S6	3.688(2)	P1–S7	2.063(3)
Cs1–S6	3.517(2)	Cs2–S7	3.622(2)	P2–S1	1.988(3)
Cs1–S6	3.732(2)	In1–S2	2.520(1)	P2–S2	2.060(3)
Cs1–S7	3.801(2)	In1–S3	2.534(1)	P2–S3	2.068(3)
Cs2–S1	3.543(2)	In1–S4	2.853(2)	P2–S4	2.074(3)
Cs2–S1	3.648(2)	In1–S4	2.834(2)		

Table S5. Selected bond angle (°) of **1**-**3**.

α-$Rb_2InP_2S_7$ (**1**)					
bond	length/Å	bond	length/Å	bond	length/Å
S1–In1–S1	83.02(4)	S5–In1–S1	90.76(4)	S2–P2–S5	109.25(8)
S2–In1–S1	88.52(4)	S5–In1–S1	75.68(4)	S5–P2–S1	104.94(7)
S2–In1–S1	76.66(4)	S5–In1–S3	95.59(4)	S7–P2–S1	111.67(8)
S2–In1–S3	98.09(4)	S5–In1–S4	96.07(4)	S7–P2–S2	113.07(8)

S2–In1–S4	94.68(4)	S3–P1–P1	103.28(9)	S7–P2–S5	111.21(8)
S2–In1–S5	160.53(4)	S4–P1–P1	102.53(9)	P2–S1–In1	84.25(5)
S3–In1–S1	167.23(4)	S4–P1–S3	106.53(7)	P2–S1–In1	85.25(5)
S3–In1–S1	87.87(4)	S6–P1–P1	109.85(8)	P2–S2–In1	92.10(5)
S4–In1–S1	169.53(4)	S6–P1–S3	115.45(8)	P1–S3–In1	97.77(6)
S4–In1–S1	90.98(4)	S6–P1–S4	117.54(8)	P1–S4–In1	97.54(6)
S4–In1–S3	99.31(4)	S2–P2–S1	106.26(7)	P2–S5–In1[1]	92.29(5)

β-$Rb_2InP_2S_7$ (**2**)

Bond Angle	Angle/°	Bond Angle	Angle/°	Bond Angle	Angle/°
S1–In1–S1	81.42(5)	S3–In1–S1	167.54(3)	S5–In2–S1	91.74(3)
S2–In1–S1	87.53(4)	S3–In1–S1	167.54(3)	S5–In2–S1	76.67(3)
S2–In1–S1	75.76(3)	S3–In1–S1	90.40(3)	S7–P1–P1	109.93(7)
S2–In1–S1	87.53(4)	S3–In1–S3	99.11(5)	S7–P1–S6	116.04(7)
S2–In1–S1	75.76(3)	S6–In2–S6	99.28(5)	S7–P1–S3	116.70(7)
S2–In1–S2	158.01(5)	S6–In2–S1	88.55(3)	S3–P1–S6	106.63(6)
S2–In1–S3	94.62(4)	S6–In2–S1	169.28(3)	S5–P2–S1	105.55(7)
S2–In1–S3	94.62(4)	S6–In2–S1	88.55(3)	S4–P2–S5	111.64(7)
S2–In1–S3	99.61(4)	S6–In2–S1	169.28(3)	S4–P2–S1	111.46(7)
S2–In1–S3	99.61(4)	S5–In2–S1	2.561(1)	S2–P2–S5	109.39(6)
S3–In1–S1	90.40(3)	S5–In2–S6	95.28(4)	S2–P2–S1	105.9(7)

$Cs_2InP_2S_7$ (**3**)

Bond Angle	Angle/°	Bond Angle	Angle/°	Bond Angle	Angle/°
S2–In1–S3	159.59(6)	S5–In1–S4	87.32(6)	S6–P2–P2	110.76(1)
S2–In1–S4	75.71(6)	S5–In1–S7	98.64(6)	S6–P2–S5	115.19(1)
S2–In1–S4	88.07(6)	S7–In1–S4	169.80(6)	S6–P2–S7	117.18(1)
S2–In1–S5	98.53(7)	S7–In1–S4	96.62(6)	S7–P2–P2	101.93(1)
S2–In1–S7	95.14(7)	S1–P1–S2	112.17(1)	S7–P2–S5	106.49(1)
S3–In1–S4	90.68(6)	S1–P1–S3	110.54(1)	P1–S2–In1	93.32(9)
S3–In1–S4	74.99(6)	S1–P1–S4	112.92(1)	P1–S3–In1	93.42(9)

S3–In1–S5	95.89(7)	S2–P1–S3	110.07(1)	P1–S4–In1	83.98(8)
S3–In1–S7	96.89(7)	S2–P1–S4	106.26(1)	P1–S4–In1	85.04(8)
S4–In1–S4	82.71(6)	S3–P1–S4	104.54(1)	P2–S5–In1	98.38(9)
S5–In1–S4	166.34(6)	S5–P2–P2	103.69(1)	P2–S7–In1	97.34(9)

2. Figures

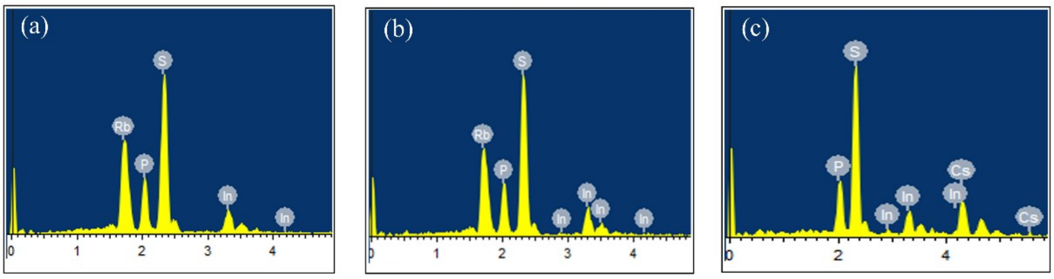

Fig. S1 EDS of **1** (a), **2** (b), and **3** (c).

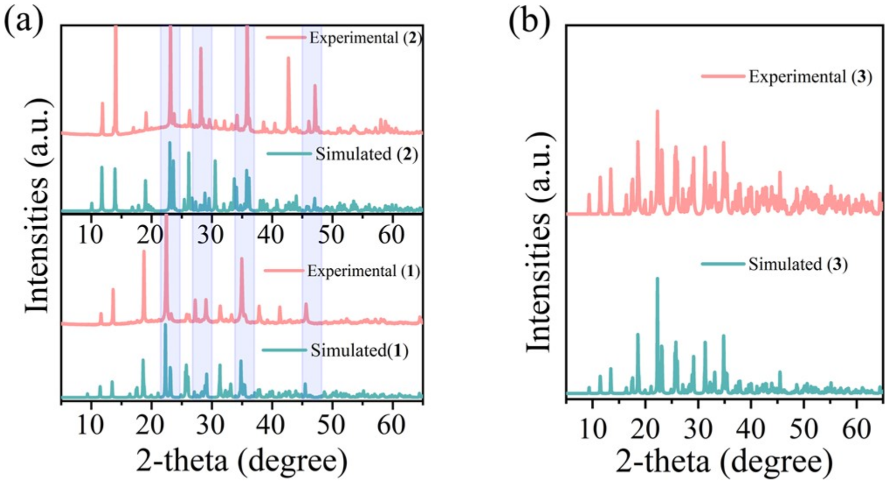

Fig. S2 Powder XRD patterns of **1**-**2** (a), and **3** (b).

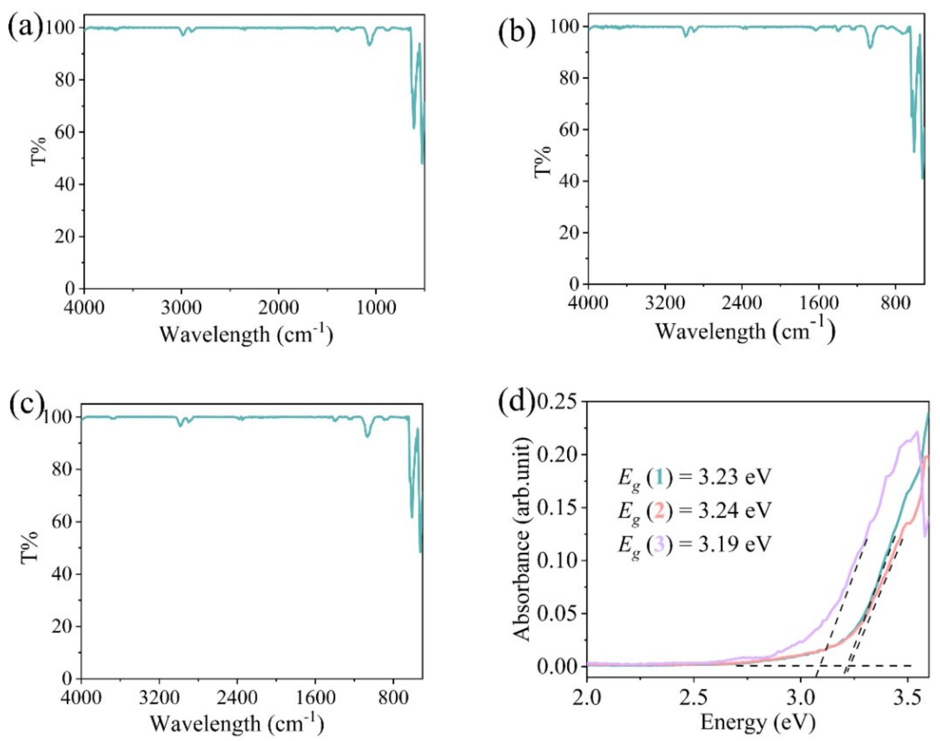

Fig. S3 (a-c) IR spectra of **1**-**3**, respectively. (d) Band gaps of **1**-**3**.

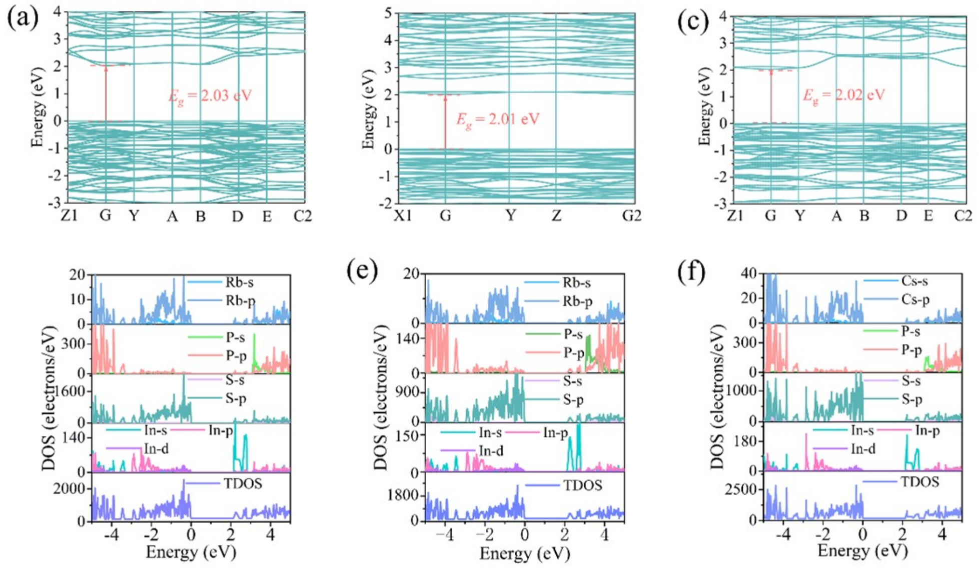

Fig. S4 Electronic band structures and the partial DOS of **1** (a, d), **2** (b, e) and **3** (c, f).

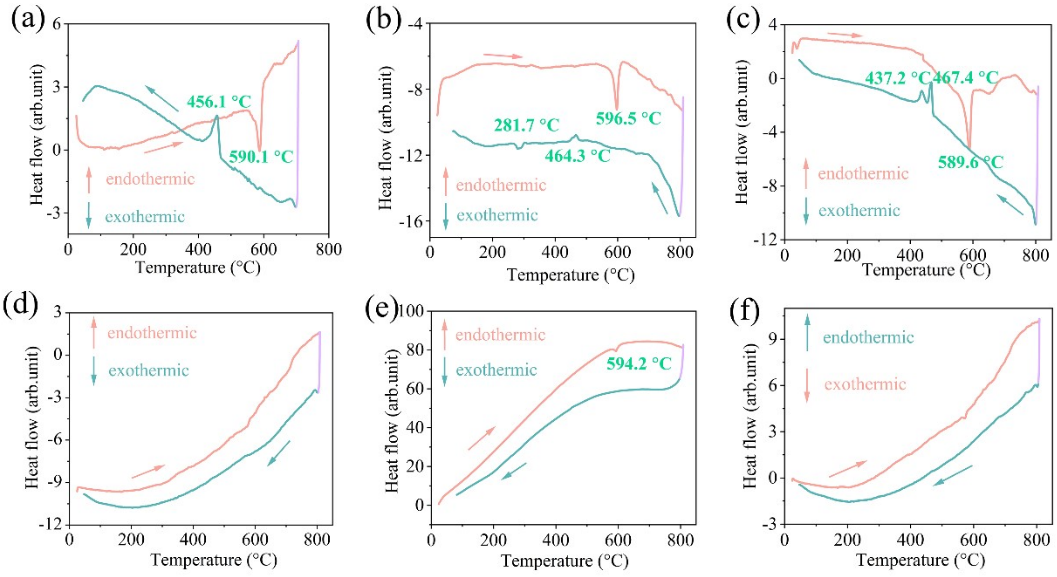

Fig. S5 The first and second DSC cycles of **1** (a, d), **2** (b, e) and **3** (c, f).

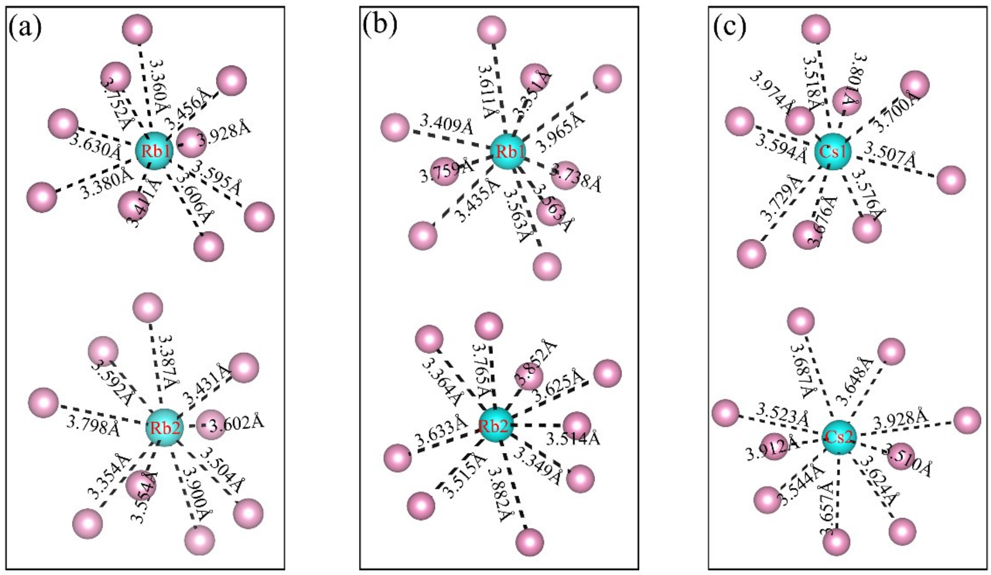

Fig. S6 Coordination environment diagram of Rb/Cs in **1**–**3**.

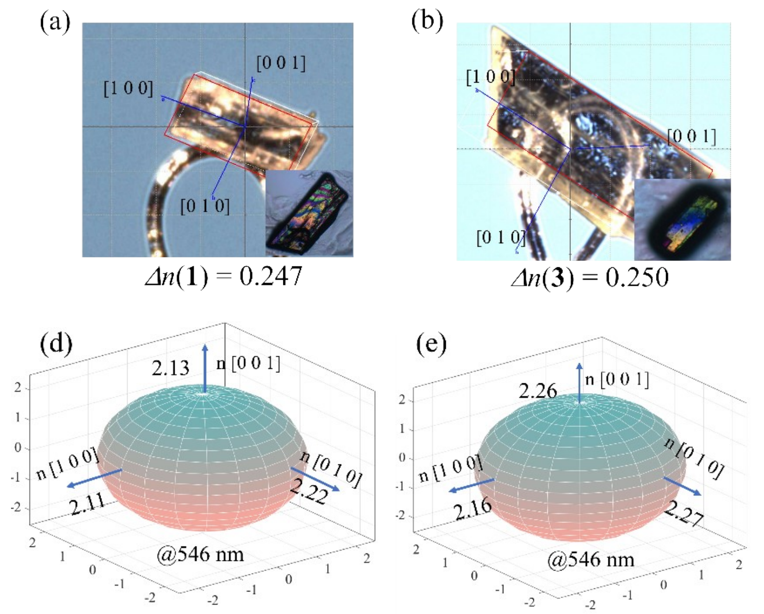

Fig. S7 The crystals orientation of **1** (a) and **3** (b) are determined by single-crystal XRD. Theoretically calculated refractive indices and birefringence of **1** (c) and **3** (d).

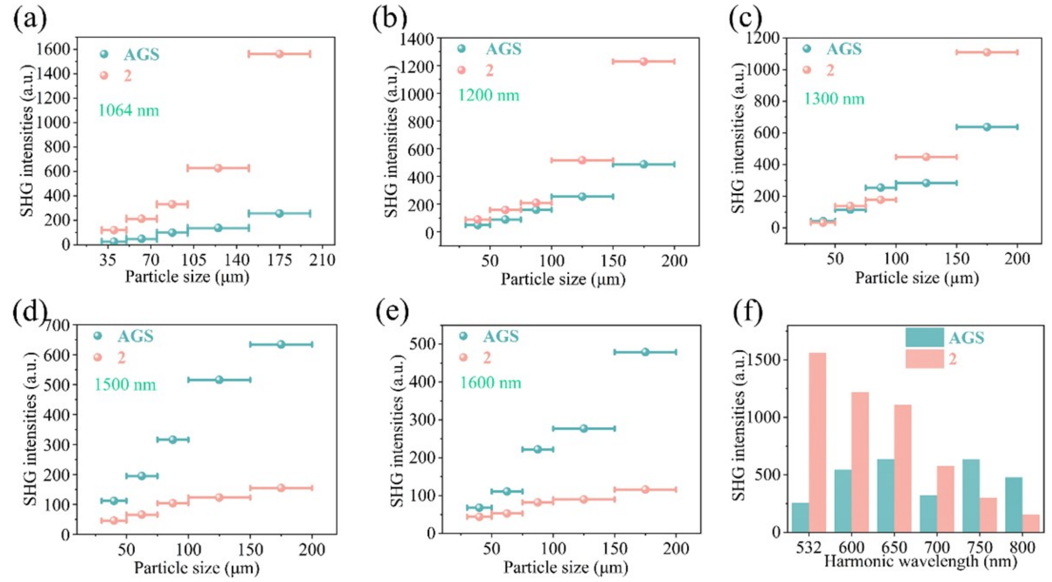

Fig. S8 Phase matchable behaviors of **2** and $AgGaS_2$ with the laser of 1064 (a), 1200 (b), 1300 (c), 1500 (d), 1600 nm (e), respectively, and broadband SHG intensities **2** and $AgGaS_2$ (f)
